# Supplementary material for: The Safety and Toxicity of Phage Therapy: A Review of Animal and Clinical Studies
Source: Viruses. 2021 Jun 29;13(7):1268. doi: 10.3390/v13071268 (PMC8310247; doi:10.3390/v13071268)
Supplement: Supplementary file 1 [file viruses-13-01268-s001.zip › viruses-1235692-supplementary.pdf]

Supplementary Figures

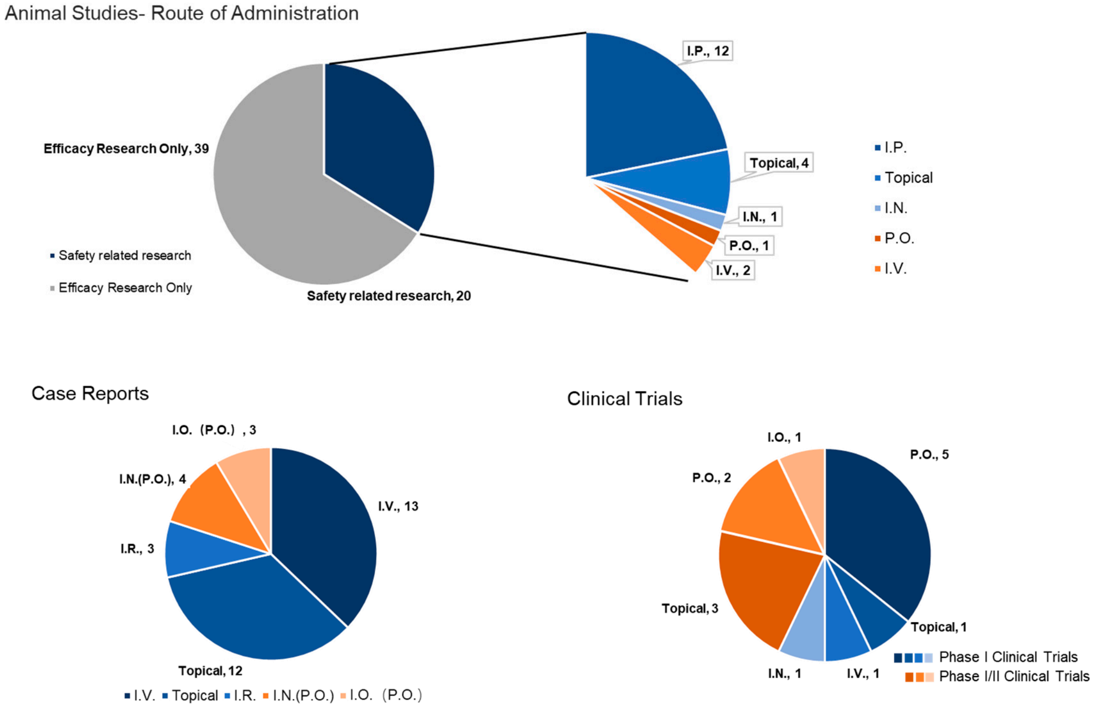

Figure S1. Animal studies and clinical studies of phage therapy 2008-2021. Our review included 59 animal studies of phage therapy. Of these, 20 examined safety measures in some capacity. Routes of phage administration included intraperitoneal (I.P.), inhalational (I.N.), topical, oral (P.O.), intravenous (I.V.). 35 case reports of phage therapy were included in humans. Of these, therapy was administered by I.V., single or combined with other administration, topical, intrarectal (I.R.), I.N., with or without P.O., Intravesical (I.O.), with or without P.O. This review also included 14 clinical trials in humans. the routes of administration included P.O., Topical, I.N., I.V. and I.O.

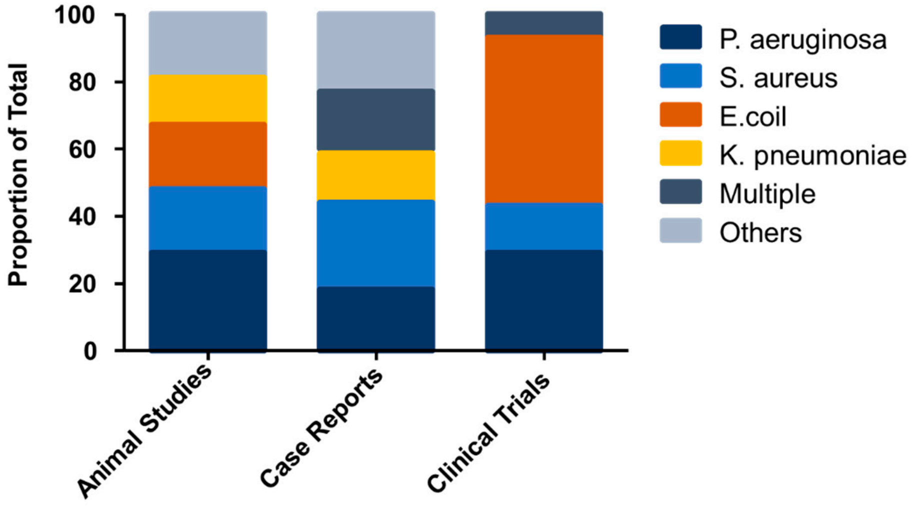

Figure S2. Pathogens targeted in phage therapy studies. The percentage of cases that targeted specific pathogens are shown for animal studies, case reports and clinical trials. The target pathogens in animal studies included *P. aeruginosa*, *S. aureus, E. coli*, *K. pneumoniae*, or other pathogens. The target pathogens in case reports were *P. aeruginosa, S. aureus, and K. pneumoniae*, multiple pathogens infection, and other pathogens. The major pathogens of interest in clinical trials were *E. coli*, *P. aeruginosa*, *S. aureus*, and multiple pathogens infection. “Other pathogens” include: *Enterococcus faecalis, Mycobacterium, Achromobacter xylosoxidans, Acinetobacter baumannii, Vibrio cholerae, Cronobacter spp.*

Supplementary Table S1. Animal Studies of Phage Therapy (6/20)

Reference	Animal model	Phage administration	Main safety outcomes
Dufour, et al. 2019 [17] France	Mice *E. coli* pneumonia	Phages 536_P1 and LM33_P I.V.	No difference in behavior and weight, Cytokine concentration in lung and blood: phage 536_P1 alone promoted a weak increase of anti-viral cytokines (INF-ϒ and IL-12) and chemokines in the lungs.
Fong, et al. 2019 [20]	Sheep *P. aeruginosa* sinuses	PA phage cocktail Topical	Phage was detected in blood, No loss of appetite, fever, or other signs of systemic illness, No significant changes in blood chemistry, SEM show no change in assess cilia.
Drilling, et al. 2017 [19]	Sheep *S. aureus* Sinusitis	Phage cocktail NOV012; Topical	No infectious phages in serum; General Health: appetite, fever, systemic illness; SEM shows no changes in cilia morphology.
Drilling, et al. 2014 [18]	Sheep *S. aureus;* sinusitis	phage cocktail (CTSA) Topical	No Phage in kidney, spleen, liver, and brain; SEM showed no change in assess cilia.
Chhibber, et al. 2008 [104]	Mice *K. Pneumoniae* pneumoniae	phage SS I.P.	No change in rectal temperature lethargy or sickness; No toxicity in mice.
Jongsoo, el.at. 2019 [105]	Mice *P. aeruginosa;* pneumoniae	phages R656, R1836 I.P.	No difference in survival; No difference in the levels of TNF-α and IL-6 in the lungs; HE showed no toxicity in lung sections.
Chang, et al. 2018 [91]	Mice *P. aeruginosa* Pneumoniae	Phage PEV20 I.N.	Level of phages in plasma was low Histopathological examination showed no lung toxicity.
Gelman, et al. 2018 [97]	Mice *E. faecalis* peritonitis	Phage EFDG1, EFLK1 I.P.	No difference in physical condition; Phage alone mildly increase the level of TNF-α; Phages alone did not result in any adverse effects.
Cheng, et al. 2017 [98]	Mice *E. faecalis* bacteremia	Phage EF-P29 I.P.	No difference in health scores or body weight between normal.
Oechslin, et al. 2016 [21] Switzerland	Rat *P. aeruginosa* Endocarditis	Phage cocktail PP1131 I.V.	Phage detected in blood, spleen, kidney, liver, lung, and brain; IL-1β, IL-6 increased plasma levels may relate to phage-induced bacterial lysis.

Galtier, et al. 2016 [45] France.	Mice<br>*E. coli*<br>intestinal colonization	Phage AL505<br>P.O.	A lower impact on microbiota composition compared to antibiotic treatment.
Jun, et al. 2014 [23]	Mice female<br>*Vibrio* parahaemolyticus	pVp-1<br>I.P. or P.O.	No changes in physical condition and survival; Titers of IgG and IgM against the phage increased by 170-fold and 50-fold, respectively. No adverse events were observed
Takemura-Uchiyama, et al. 2014 [106]	Mice<br>*S. aureus*<br>septicaemia	Phage S13'<br>I.P.;	No unusual behavior or deaths were observed.
Osanai, et.al. 2012 [107]	Mice<br>*K. Pneumoniae*<br>bacteremia	Phage cocktail<br>I.P.	Phage shows in blood samples; No fever or general lethargy 30 days after injection; no adverse effects.
Pouillot, et al. 2012 [108] France	Rat<br>*E. coli*<br>Sepsis and Meningitis	Phage S242<br>I.P. or s.c.	High concentration in blood, spleen and kidney, low in brain at 2-24 h; no difference in weight gain; no sign of toxicity.
Ľubomíra Tóthová et al. 2011 [109] Slovakia	Mice<br>*Cronobacter*<br>UTI	Cronobacter-specifc phage<br>I.P.	No adverse effects were reported.
Hung, et al. 2011 [110]	Mice<br>*K. pneumoniae*<br>Bacteremia	bacteriophage NK-5<br>I.P. vs. P.O.	No difference in survival rate; phage alone resulted in no elevation of AST and ALT,TNF-α， IL-6, MCP-1, IFN-γ, IL-10, and IL-12 p70 levels; No death or adverse effect.
Hawkins, et al. 2010 [111] UK	Dog<br>*P. aeruginosa*<br>otitis	Cocktail with Six phages<br>Topical	No treatment related inflammation was detected; no related adverse events.
Sunagar, et al. 2010 [22]	Mice<br>*S. aureus*<br>bacteremia	Phage GRCS<br>I.P.	No anaphylactic reactions or changes in core body temperature; titers of IgG and IgM against the phage increased above the background by 2500-fold and 100-fold respectively. No other adverse events.
Nishikawa, et al. 2008 [112]	Mice<br>*E. coli*<br>UTI	phages T4 and KEP10<br>I.P.	No adverse effects according the physical exam and survival data

Supplementary Table S2. Case Reports of Phage Therapy (16/ 35)

Reference	Case	Phage administration	Main safety outcomes
Lebeaux et al., 2021 [113] Belgium	12-year-old male lung-transplanted cystic fibrosis *A. xylosoxidans*	phage against *A. xylosoxidans;* Inhale	Clinical tolerance was perfect No details.
Ferry et al., 2020 [114] France	Three case with S. aureus prosthetic joint infection	PP1493, PP1815, PP1957 Topical	No safety monitoring.
Bao et al., 2020 [115]	63-year-old female UTI with *K. pneumoniae* infection	phages against *K. pneumoniae*. Topical	No adverse occurred No details.
Cano, et al.2020 [116]	62-year-old male; *K. pneumoniae* prosthetic knee infection	KpJH46Φ2 I.V.	No treatment-related adverse effects and remained asymptomatic 34 weeks after completing treatment.
Rostkowska, et al. 2020 [117] Poland	60-year-old male; *K. pneumoniae* UTI	phage against *K. pneumoniae;* Intrarectal	No adverse reactions or intolerance reported.
Doub, et al. 2020 [24]	72-year-old male; *S. aureus* prosthetic joint infection.	SaGR51’1; I.A. + I.V.	Albumin decreased, therapy was stopped due to transaminitis.
Rubalskii et al.2020 [118] Germany	Case series of Cardiothoracic Surgery Related Infections	Multiple phages; Topical or I.N.	No adverse events reported.
Gainey et al.2020 [119]	10-year-old female *Achromobacter* CF	Phage Ax2CJ45Φ2 I.V.	No adverse events reported.
Aslam et al.2019 [120]	3 lung transplant patients *P. aeruginosa or B. dolosa*	AB-PA01; Navy phage cocktail; BdPF16phi4281; I.V.	Clinical and laboratory parameters were tested. No phage-related adverse events were identified
Nir-Paz el at. 2019 [121] Israel (associated state of EU)	A 42-year-old male *Acinetobacter baumannii* and *K. pneumoniae* bone infection	/ I.V.	No adverse events related to phage were noted clinically or on laboratory monitoring (liver function tests, complete blood counts, electrolytes).
Tkhilaishvili et al. 2019 [122] Germany	80-year-old female *P. aeruginosa* Joint Infection	/ Topical	No adverse events reported.
Onsea et al.2019 [123]	Case series	BFC1 cocktail Pyo bacteriophage	Clinical status was evaluated daily. No adverse events reported.

Belgium	Musculoskeletal Infections	Topical	
Corbellino et al.2019 [124] Switzerland	57-year-old *K. pneumoniae* gut infection	oral + I.R.	No adverse events reported.
Susan, et al. 2019 [125]	77-year-old female; *P. aeruginosa;* pneumonia	AB-PA01; I.V.+ Inhale;	Well-tolerated with no adverse events detected either during therapy or subsequently.
Gilbey, et al. 2019 [89]	65-year-old male *S. aureus* Endocarditis	AB-SA01 I.V.	No fevers, tachycardia, hypotension or rashes were detected after phage infusions and no adverse sequelae were attributable to the therapy.
Law, et al. 2019 [90]	26-year-old female *P. aeruginosa* CF	AB-PA01 I.V.	No adverse events were noted clinically or by lab exam
RM, et al. 2019 [28]	15-year-old *Mycobacterium abscessus* CF	BPs33ΔHTH-HRM10 and ZoeJΔ45 I.V. + Topical	Sera showed weak phage-neutralization antibody and cytokine responses. Diaphoresis and flushing but no fever or changes. No adverse reactions to phage administration.
Kuipers et al. 2019 [126] Netherlands	58-year-old male Klebsiella pneumoniae UTI	/ oral + I.O.	No adverse events occurred.
Duplessis, et al. 2018 [10]	A 2-year-old male *P aeruginosa* bacteremia;	phage cocktail I.V.	Without identified host humoral responses targeting phage; Phage was withheld for decompensation concerning for anaphylaxis.
LaVergne et al. 2018 [27]	77-year-old male *A. baumanii* brain injury surgical related infection	*A. baumani* phage I.V.	Brief episode hypotension after the first dose.
Ferry et al.2018 [127] France	80-year-old female *S. aureus* prosthetic joint infection	S. aureus phage Topical	No adverse events reported to phage therapy.
Fish, et al. 2018 [128]	63-year-old female *S. aureus* Osteomyelitis	Sb-1; Topical	No safety monitoring.
Ferry, et al. 2018 [85] France	60-year-old male *S. aureus* bone and joint infection	Phage cocktail Topical	No safety monitoring.

Hoyle, et al. 2018 [129]	17-years-old female *Achromobacter xylosoxidans* CF	phage cocktail Inhale + oral	No safety monitoring.
Chan, et al. 2018 [130]	76-year old male *P. aeruginosa* aorto-cutaneous fistula infection	phage OMKO1 Instill	The patient had no complaints with stable vital signs and had laboratory values within normal limits.
Ujmajuridze et al. 2018 [26] Switzerland	UTI case serial with different pathogen	phage cocktail Pyo Intravesical (I.O.)	One patient experienced sudden fever and chills on the third day of PT. No bacteriophage-associated adverse events have been detected.
Schooley, et al. 2017 [25]	68-year old female A. baumannii Pancreatitis	Cocktail with 9 phages I.V. + Instill	Phage neutralization antibody appeared in plasma (in vitro); PT was withheld for increased pressor requirements two days following initiation.
Zhvania, et al. 2017 [131] Georgia	16-year-old male; *S. aureus* skin infection	Sb1, Topical,	No adverse reactions.
Jennes, et al. 2017 [132] Belgium	61-year-old male *P. aeruginosa* Septicaemia;	Phage cocktail BFC1 I.V. + Instill	No adverse events, clinical abnormalities or changes in lab test related to phages
Fish et al. 2016 [133]	Case series, *S. aureus* diabetic foot ulcer	Sb-1; Topical	No adverse events reported
Fadlallah et al. 2015 [134] France	65-year-old female *S. aureus* corneal abscess	phage SATA-8505 Topical	No adverse events reported
Rose et al. 2014 [135] Belgium	burn wounds case serial with *S. aureus* and *P. aeruginosa*	phage cocktail BFC-1; Topical	No adverse events, clinical abnormalities or changes in laboratory test related to the application of phages
Khawaldeh, et al. 2011 [136]	67-year-old female *P. aeruginosa* UTI	Pyophage Instill	No adverse events reported.
Kvachadze, et al. 2011[137]	7-year-old female *S. aureus* and *P. aeruginosa* CF	Sb-1 and Pyophage Nebulizer	No adverse events reported.
Letkiewicz,el.at.2009 [138] Poland	Case series; *E. faecalis* chronic prostatitis	Coliphage Intrarectal	No change significantly in the function of liver, pancreas, kidney, and bone marrow immune function

Supplementary Table S3. Clinical Trials of Phage Therapy (7/14)

Reference	Trial	Phage administration	Main safety outcomes
Leitner et al., 2020 [99] Switzerland	Phase I/II clinical trial (UTI)	Pyophage Intravesical (I.O.)	Safety assessment included frequency and severity of adverse events during the treatment period according to the （CTCAE） v4.0 as grade 1 to 5.
Grubb et al., 2020 [44]	Phase I/II clinical trial (gastrointestinal distress)	PreforPro Oral	glucose, BUN, creatinine (CRE), creatinine kinase (CK), NA+, K+, Cl- and C-reactive protein; a daily stool log, and gut microbial populations.
Petrovic Fabijan et al. 2020 [139]	Phase I clinical trial (endocarditis, sepsis)	AB-SA01 I.V.	The vital signs, clinical, haematological and blood biochemical parameters. The local adverse effects and systemic adverse reactions and evidence of renal or hepatic dysfunction. No adverse reactions were reported.
Ooi et al. 2019 [32]	Phase I clinical trial (Rhinosinusitis)	AB-SA01; Topical	Vital signs, physical examinations, clinical laboratory test results, and adverse events. Intranasal phage treatment was well tolerated, with no serious adverse events or deaths reported.
Febvre, et al. 2019 [30]	Phase I clinical trial	Coliphage cocktail Oral;	a small but significant decrease in circulating IL-4 according the system inflammatory examination.
Gindin, et al. 2019 [96]	Phase I clinical trail	coliphages Oral	No difference in liver and kidney function and metabolic parameters;<br>No adverse events observed by self-report or clinical exam.
McCallin, et al. 2018 [31] Belgium	Phase I clinical trial	Pyophage cocktail; Oral or Nasal;	No difference in physical conditions; No different in clinical lab exam. No adverse events related to phage administration. The body temperature showed more fluctuations, but fever was only rarely observed and did not exceed 38 °C.
Sarker, et al. 2017 [47] Switzerland	Phase I clinical trial	Two coliphage cocktails Oral	No viable coliphage in the serum; No difference in physical conditions; No difference in clinical lab exam. No serum was positive for LPS; No antibody increasing to LPS or phage. No adverse effects.

McCallin, et al. 2013 [46] Switzerland	Phase I clinical trial	coliphage cocktail Oral	No safety issues reported according the physical data, clinical lab exam and no phage or phage antibody in serum detected. No adverse effects Have no impact on fecal microbiota composition.
Sarker, et al. 2012 [48]	Phase I clinical trial	T4-like phage cocktail Oral	No subjects complain; No safety issues reported according the vital sign, clinical lab exam, No adverse effects and impact on fecal microbiota composition.
Rhoads, et.al 2009 [140]	Phase I clinical trial (venous leg ulcers)	WPP-201 phage cocktail Topical	No safety issues reported according the vital sign, lab exam.
Jault, et al. 2019 [141] France	Phase I/II clinical trial (burn patient)	phages cocktail PP1131 Topical	No substantially different in vital sign, physical examinations
Sarker, et al. 2016 [142] Switzerland	Phase I/II clinical trial (Acute bacterial diarrhea)	Two coliphages cocktails Oral	No changes in physical examinations and vital sign. No adverse events.
Wright. A. el, at. 2009 [95] UK	Phase I/II clinical trial (chronic otitis)	Biophage-PA Cocktail; Topical	No reportable side effects from patient, and no evidence of local or systemic toxicity.
